# Transcriptional profiling of trait deterioration in the insect pathogenic nematode *Heterorhabditis bacteriophora*

**DOI:** 10.1186/1471-2164-10-609

**Published:** 2009-12-15

**Authors:** Bishwo N Adhikari, Chin-Yo Lin, Xiaodong Bai, Todd A Ciche, Parwinder S Grewal, Adler R Dillman, John M Chaston, David I Shapiro-Ilan, Anwar L Bilgrami, Randy Gaugler, Paul W Sternberg, Byron J Adams

**Affiliations:** 1 Department of Biology and Evolutionary Ecology Laboratories, Brigham Young University, Provo, UT 84602, USA; 2 Department of Microbiology and Molecular Biology, Brigham Young University, Provo, UT 84602, USA; 3 Department of Entomology, The Ohio State University-OARDC, Wooster, OH 44691, USA; 4 Department of Microbiology and Molecular Genetics, Michigan State University, 2215 Biomedical Physical Sciences Building, East Lansing, MI 48824, USA; 5 Division of Biology, California Institute of Technology, 1200 E. California Blvd., Pasadena, CA 91125, USA; 6 Department of Bacteriology, University of Wisconsin, Madison, WI 53076, USA; 7 USDA-ARS, Southeastern Fruit and Nut Research Laboratory, 21 Dunbar Road, Byron, GA 31008, USA; 8 Department of Entomology, Rutgers University, New Brunswick, NJ 08901, USA

## Abstract

**Background:**

The success of a biological control agent depends on key traits, particularly reproductive potential, environmental tolerance, and ability to be cultured. These traits can deteriorate rapidly when the biological control agent is reared in culture. Trait deterioration under laboratory conditions has been widely documented in the entomopathogenic nematode (EPN) *Heterorhabditis bacteriophora *(*Hb*) but the specific mechanisms behind these genetic processes remain unclear. This research investigates the molecular mechanisms of trait deterioration of two experimental lines of *Hb*, an inbred line (L5M) and its original parental line (OHB). We generated transcriptional profiles of two experimental lines of *Hb*, identified the differentially expressed genes (DEGs) and validated their differential expression in the deteriorated line.

**Results:**

An expression profiling study was performed between experimental lines L5M and OHB of *Hb *with probes for 15,220 ESTs from the *Hb *transcriptome. Microarray analysis showed 1,185 DEGs comprising of 469 down- and 716 up-regulated genes in trait deteriorated nematodes. Analysis of the DEGs showed that trait deterioration involves massive changes of the transcripts encoding enzymes involved in metabolism, signal transduction, virulence and longevity. We observed a pattern of reduced expression of enzymes related to primary metabolic processes and induced secondary metabolism. Expression of sixteen DEGs in trait deteriorated nematodes was validated by quantitative reverse transcription-PCR (qRT-PCR) which revealed similar expression kinetics for all the genes tested as shown by microarray.

**Conclusion:**

As the most closely related major entomopathogen to *C. elegans*, *Hb *provides an attractive near-term application for using a model organism to better understand interspecies interactions and to enhance our understanding of the mechanisms underlying trait deterioration in biological control agents. This information could also be used to improve the beneficial traits of biological control agents and better understand fundamental aspects of nematode parasitism and mutualism.

## Background

Biological control using predators, parasitoids, or pathogens, can be an effective alternative for management of arthropod pests [1,2]. In contrast to chemical insecticides, biological control agents are generally not harmful to humans or the environment, and have minimal or negligible potential to cause resistance or harm to non-target organisms. The success of a biological control agent depends on key traits, particularly compatibility with the target pest, reproductive potential, host-finding ability, environmental tolerance, and ability to be cultured. These traits, however, can deteriorate rapidly, and substantially when a biological control agent is isolated from nature and reared in the laboratory, or mass-produced for commercial purposes [3-5]. Genetic and non-genetic processes may be responsible for trait deterioration in laboratory-cultured biological control agents. Loss of genetic variation due to inadvertent selection [5,6], exposure of deleterious recessive genes, increased homozygosity because of inbreeding [3], and disproportionate representation of genotypes in successive generations due to genetic drift [3] during sub-culturing can impair the effectiveness of biological control agents. Trait deterioration may also result from non-genetic factors such as poor nutrition and disease [4].

Entomopathogenic nematodes (EPNs) in the families Heterorhabditidae (Strongyloidea) and Steinernematidae (Strongyloidoidea sensu) [5] are biological control agents that serve as exceptional models for the study of parasitism, pathogenicity, and symbiosis [3-5]. These nematodes form mutualistic symbioses with insect pathogenic bacteria in the family Enterobacteriaceae: heterorhabditids are associated with *Photorhabdus *and steinernematids with *Xenorhabdus*, respectively [7]. The infective juveniles (IJs) or dauer (enduring) juveniles persist in soil in search of a suitable insect host [8]. Following entry through the cuticle or natural body openings, the IJs release the symbiotic bacteria into the insect hemocoel, which rapidly kill the host, usually within 24-48 h [9]. Nematodes feed on symbiotic bacteria and digested host tissues, complete 1-3 generations in the host cadaver, and as food resources are depleted new IJs are produced which disperse in search of new hosts. In the laboratory, each partner can be cultured separately, but when combined they present a high degree of specificity [7]. These EPNs are cultured for experimental or commercial purpose using in vivo or in vitro methods [10].

Deterioration of traits essential for biological control has been recognized in diverse biological control agents [4,11-13] including EPNs [14,15]. Trait deterioration under laboratory conditions has been widely documented in various biological control agents including predators, parasitoids and pathogens [3]. Similarly, microbial control agents such as viruses (e.g. baculoviruses), bacteria (e.g. *Bacillus thuringiensis*) and fungi (e.g. *Beauveria bassiana*) have been reported to lose virulence when sub-cultured in the laboratory [2,11,16]. Previous research has shown that traits can deteriorate rapidly in EPNs [14,15,17] and in their symbiotic bacteria [18]. Shapiro et al. [19] reported a reduction in heat tolerance of *Heterorhabditis bacteriophora *(*Hb*) under laboratory conditions. Similarly, Wang and Grewal [15] reported rapid deterioration in environmental tolerance and fecundity for *Hb *during laboratory maintenance. Bilgrami et al. [14] showed that genetic factors play a significant role in the deterioration process; however, the specific mechanisms behind these genetic processes remain unclear. Additionally, physiological or biochemical effects such as nutritional factors may also contribute to trait deterioration. Therefore, establishing stability in beneficial traits requires an understanding of the mechanisms involved in trait deterioration, specifically, the molecular genetic processes. This research investigates the molecular mechanisms of trait deterioration of two experimental lines of an EPN, an inbred line (L5M) (created by sub-culturing different experimental lines of the nematode-bacterium complex over 20 passages in insect hosts) and its original parental line (OHB). These lines differed in their virulence, heat tolerance and fecundity [14]. We generated transcriptional profiles of the two experimental lines of EPN, then identified and validated the genes that were differentially expressed (DE) in the deteriorated line.

## Results

To identify genes associated with trait deterioration in the entomopathogenic nematode *Hb*, an expression profiling study was performed using custom Roche NimbleGen expression arrays with probes for 15,220 ESTs from the *Hb *transcriptome. To identify the genes involved in trait deterioration, expression was analyzed between two experimental lines of *Hb*; L5M and OHB. Four biological replicates of each line were used in hybridization experiments, allowing us to identify putative genes involved in the deterioration of important traits in *Hb*.

### Microarray analysis

**Figure 1:**
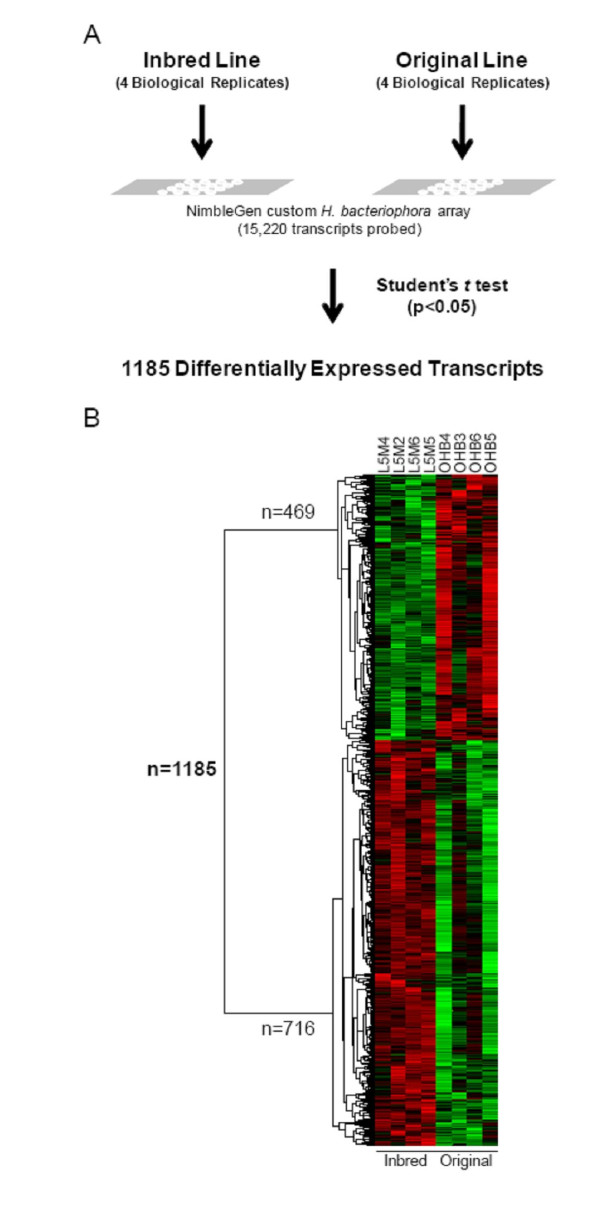
**Summary of microarray analysis and results**. **(A) **Experimental design of the microarray experiments included four biological replicates from both the inbred (L5M4, 2, 6 and 5) and the original parental line (OHB4, 3, 6 and 5). RNA from each replicate sample was fluorescently labeled and hybridized to a custom microarray containing probes for 15,220 *Heterorhabditis bacteriophora *transcripts identified in an EST library. Statistical analysis of microarray data identified 1,185 transcripts with significant (p < 0.05) differential expression between the two lines. (**B) **Clustergram of the profiles of the 1,185 differentially expressed transcripts (rows) in the eight microarray experiments (columns). Higher expression levels, relative to the mean expression levels for a given transcript, are indicated by red features and lower expression levels are indicated in green. Differences in intensity reflect gradations of over- or under-expression. Transcripts were hierarchically clustered into those with similar profiles.

**Figure 2:**
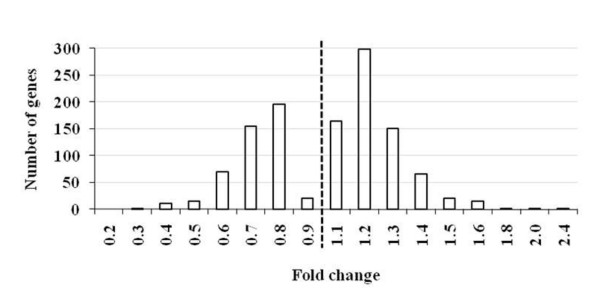
**Distribution of fold change expression level for genes differentially expressed in trait deteriorated nematodes**. The histogram show the distribution of fold change for genes differentially expressed in trait deteriorated line of nematode. The differentially expressed genes were identified by using *P*-value less than 0.05.

Microarray analysis showed 1,185 genes differentially expressed between L5M and OHB. Of those differentially expressed genes (DEGs) 469 (39.58%) were down-regulated and 716 (60.42%) were up-regulated at *P *< 0.05 (Figure 1). Many microarray studies have attempted to identify DEGs by using two-fold as threshold while in our study a two-fold cutoff would have eliminated all but 2 DEGs. The fold change in gene expression was from 0.92 to 0.38 (down-regulated) and from 1.07 to 2.45 fold (up-regulated) while considering one (1.0) as the baseline expression level. Of the total down-regulated genes, 27 (2.28%) DEGs have 0.5-0.2 fold change in expression while 442 (37.30%) DEGs have 0.9-0.6 fold change in gene expression. Among up-regulated genes, 39 (3.29%) have expression level of 1.5-2.4 fold while 677 (57.13%) have expression level of 1.1-1.4 fold (Figure 2). The average down- and up-regulation was 0.75 and 1.28 fold changes respectively. Our analysis suggests that modest expression changes involving a large number of genes are associated with trait deterioration.

### Putative functional identification of differentially expressed ESTs

**Figure 3:**
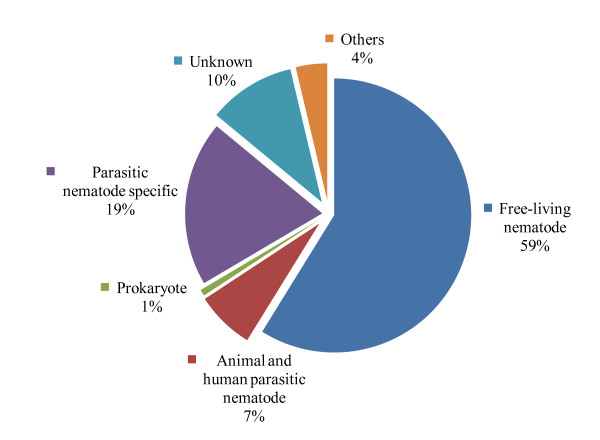
**Categories of organisms with significant protein matches to distinct *Heterorhabditis bacteriophora *ESTs**. The percentage was calculated considering the total number of *Heterorhabditis bacteriophora *differentially expressed ESTs having significant matches (E value < 1e-5) as 100%.

In order to assess the putative identities, all differentially expressed ESTs (1,185) were subjected to BLASTx sequence similarity searches against GenBank's nr database and WormBase [20] database (WS200) consisting of extensively curated *Caenorhabditis elegans *proteins. Of the 1,185 DEGs, 89% (1,063) had significant matches (E value cutoff 1e-5) to proteins in GenBank's nr database; most of the best matches (95%) were to nematode proteins. A small portion (less than 1%) of the best matches was to prokaryotic proteins. The remaining 4% of the best matches were to other eukaryotes, including humans, insects, and plants (Figure 3). The remaining 122 DEGs had no match with any sequences in the GenBank nr database. The similarity search against the *C. elegans*-specific database WS200 showed 58% (n = 698) of the DEGs had significant matches (E value cutoff 1e-5) to *C. elegans *proteins. In order to identify parasitic nematode-specific DEGs during trait deterioration, a comparison of ESTs to other nematode EST sequences from GenBank was performed. Of the 1,185 DEGs, 7% (n = 82) matched those of animal and human parasitic nematodes (AHPNs) while less than 1% (n = 10) of the ESTs matched other parasitic nematode ESTs. Of the total ESTs, 231 matched parasitic nematode-specific ESTs but did not match AHPNs or other parasitic nematode ESTs which are designated as parasitic nematode-specific (PNS) or *Hb*-specific ESTs (Figure 3). We identified 114 genes that exhibited *C. elegans *RNAi phenotypes (selected phenotypes are listed in Additional file 1).

As an important starting point in the prediction of molecules that are secreted or excreted in or during host-parasite interaction, we identified 101 putatively secreted proteins representing a non-redundant catalogue of *Hb *molecules (Additional file 2). Examples of such proteins are cysteine proteinase, aspartyl protease, diadenosine tetraphosphatase, Hsp70-interacting protein and calumenin (calcium-binding protein). In the present data set (= 1,185 DEGs), we identified 101 (9%) putatively secreted proteins with homologies to diverse organisms. Of these, 14 (14%) sequences had no significant similarity to any sequence available in current databases, whereas 87 (86%) had homologues in nematodes and other organisms, with 60 (59%) *C. elegans *and/or *C. briggsae *matches, 11 (11%) AHPNs like *Brugia malayi*, *Ostertagia ostertagi *and *Ancylostoma ceylanicum*, 11 (11%) from eukaryotes other than nematodes (fungi, plants, insects and animals), and 3 (3%) from prokaryotes (like *Burkholderia mallei *and *Neisseria lactamica*), and 2 (2%) other eukaryotes (parasites and vector agents).

### Annotation and gene ontology analysis of differentially expressed ESTs

**Figure 4:**
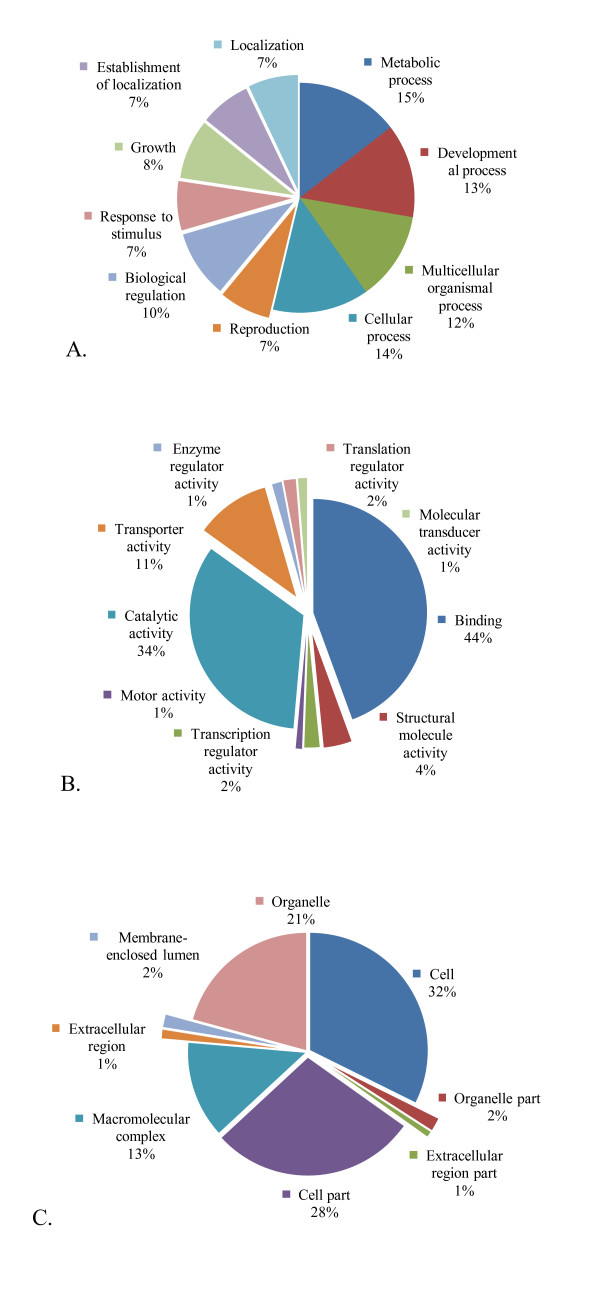
**Percent representation of gene ontology (GO) mappings for *Heterorhabditis bacteriophora *differentially expressed genes**. Distribution of (A) Molecular functions; (B) Cellular components; and (C) Biological process categories based on gene ontology for *Heterorhabditis bacteriophora *differentially expressed ESTs. Analysis was based on GO terms assigned to 712 differentially expressed sequences. Note that individual GO categories can have multiple mappings.

ESTs of DEGs were annotated into different functional groups using Gene Ontology (GO) and mapped to different pathways using the Kyoto encyclopedia of genes and genomes (KEGG) [21]. Gene Ontology [22] has been used widely to predict gene function and classification. GO provides a dynamic vocabulary and hierarchy that unifies descriptions of biological, cellular and molecular functions across genomes. We used Blast2GO [23], a sequence-based tool to assign GO terms, extracting them for each BLAST hit obtained by mapping to extant annotation associations. We found that of the 1,185 DEGs, 28% (n = 334) could be functionally assigned to biological processes (n = 548), cellular components (n = 417) and molecular functions (n = 537) with total of 1,141 GO terms (Figure 4). Amongst the most common GO categories representing biological processes were: metabolic process (n = 315), developmental process (n = 288), multicellular organismal process (n = 271), cellular process (n = 295) and growth (n = 184). Under cellular components, the higher GO term was for cell (n = 409), cell part (n = 359), organelle (n = 263) and macromolecular complex (n = 168). The largest GO terms in molecular functions were for binding (n = 410) followed by catalytic activity (n = 309), transporter activity (n = 98), structural molecule activity (n = 37) and transcription regulator activity (n = 20) (Figure 4).

Biochemical functionality was predicted by mapping all 1,185 differentially expressed ESTs to pathways, using Blast2GO [23], with an E-value cut-off of 1e-5. Enzyme commission (EC) numbers were used to appraise which sequences pertained to a specific pathway. A total of 19% (n = 224) of the sequences were mapped to 150 KEGG pathways, with 61% (138) of the sequences representing metabolic enzymes characterized by unique EC numbers (Additional file 3). The metabolism group was dominated by 'energy' followed by 'carbohydrate' and 'amino acid' metabolism. The complete listing of metabolic enzymes is shown in Additional file 4. Metabolic molecules involved in neurodegenerative disease (n = 21) and signal transduction mechanisms (n = 14) (complete list in Table 1) had high representation amongst the sequences mapped to KEGG pathways. Enzymes involved in cellular processes and cell communication were least represented in KEGG pathways (Additional file 3). The most represented enzymes were cytochrome c oxidase (n = 43) followed by H+-transporting two sector ATPase (n = 11), H+-exporting ATPase (n = 10), protein disulfide-isomerase (n = 10) and protein-glutamine gamma-glutamyltransferase (n = 10).

**Table 1:** Signal transduction-related transcripts exhibiting differential expression between original parental line (OHB) and trait-deteriorated (inbred) line (L5M) in *Heterorhabditis bacteriophora*.

GenBank Accession number	Enzyme	Signalling pathway	NrSeq^€^	Fold change	*P*-value^£^
ES740228	Stress-induced-phosphoprotein 1	Calcium signalling	1	1.32	0.021
		Wnt signalling	1		
		VEGF signalling	1		
		TGF-beta signalling	1		
		MAPK signalling	1		
EX009882	Protein-tyrosine kinase	Calcium signalling	1	0.87	0.044
		Jak-STAT signalling	1		
		ErbB signalling	1		
		VEGF signalling	1		
EX009150	Phospholipase C beta homolog	Calcium signalling	1	1.26	0.043
		Wnt signalling	1		
		VEGF signalling	1		
		ErbB signalling	1		
		Phosphatidylinositol sign	1		
EX009598	NADPH-cytochrome P450	Calcium signalling	1	0.88	0.019
ES741918	Sodium/Potassium ATPase	Calcium signalling	1	0.64	0.039
		Two component system	1		
EX007037	Peptidylprolyl isomerase	Calcium signalling	1	0.87	0.013
ES740428	Ubiquitin conjugating enzyme	Jak-STAT signalling	1	1.54	0.006
		ErbB signalling	1		
		Wnt signalling	1		
		TGF-beta signalling	1		
ES743969	Ubiquitin conjugating enzyme	Jak-STAT signalling	1	1.52	0.011
		Wnt signalling	1		
		ErbB signalling	1		
		TGF-beta signalling	1		
ES411663	Cyclophilin-1	Jak-STAT signalling	1	1.25	0.043
		MAPK signalling	1		
ES740900	Glycogen synthase kinase 3 beta	ErbB signalling	1	0.80	0.014
		Wnt signalling	1		
		Hedgehog signalling	1		
EX007896	DNA-directed RNA polymerase	Two component system	1	0.79	0.011
EX012170	Glutamine synthetase	Two component system	1	0.61	0.046
XP849696	K+-transporting ATPase	Two component system	1	1.35	0.011
		Two component system	1		
EDP31097	Protein-tyrosine-phosphatase	MAPK signalling	1	1.25	0.043
		Jak-STAT signalling	2		
		TGF-beta signalling	1		

^€^Number of differentially expressed sequences mapped to a given signalling pathway.

^£^According to student t-test; *P *< 0.05.

### Validation of differential expression with quantitative reverse transcription-PCR

**Figure 5:**
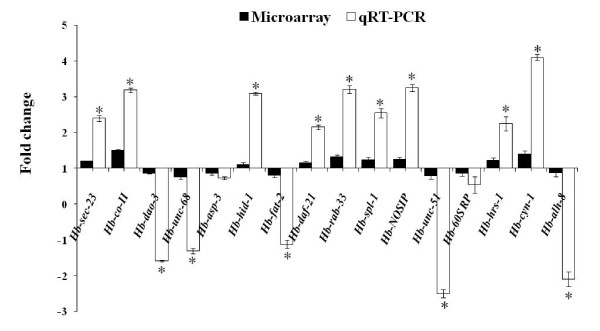
**Comparison of expression of representative genes selected from microarray data with qRT-PCR**. Comparison of fold change values from microarray data with expression ratios calculated from qRT-PCR. Values were determined using qRT-PCR and represents relative expression of genes between L5M and OHB. The relative expression of the target gene (*Hb-sec-23*: Yeast sec homolog, *Hb-co-II*: Cytochrome c oxidase II, *Hb-dao-3*: Dauer or aging adult overexpression, *Hb-unc-68*: Uncoordinated, *Hb-asp-3*: Aspartyl protease, *Hb-hid-1*: High temperature induced dauer formation, *Hb-fat-2*: Fatty acid desaturase, *Hb-daf-21*: Abnormal dauer formation, *Hb-rab-33*: RAB family member, *Hb-spl-1*: Sphingosine-1-phosphate lyase) normalized to *Hb-18s*:18S rRNA and relative to the expression of control. Bars represent standard errors calculated from 4 replicates of each experiment. *Significant difference (*P *< 0.05) between qRT-PCR and microarray data.

**Figure 6:**
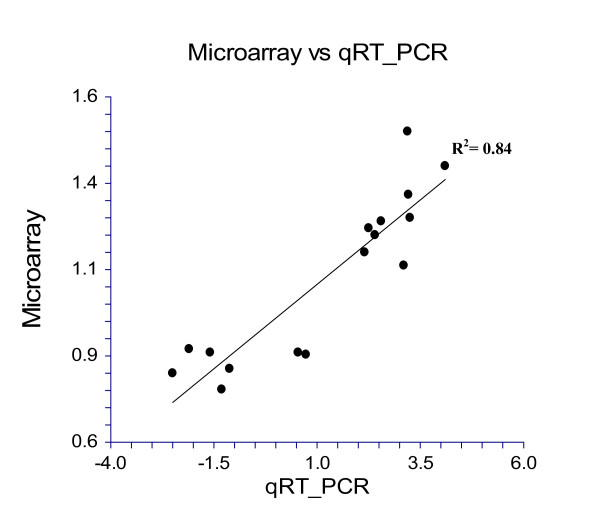
**Correlation between the fold change values from microarray and the expression ratios calculated from qRT-PCR presented as level of gene expression**. Correlation coefficient between the fold change values from microarray and qRT-PCR.

We selected sixteen genes for validation of the microarray data by quantitative reverse transcription-PCR (qRT-PCR) using gene-specific primers (Additional file 5). Four biological replicates of each line were used to determine the effect on metabolism, stress, life span and dauer development-associated candidate gene expression. The values indicated in the bar diagram in Figure 5 represent the fold change in the target gene, normalized to 18S ribosomal RNA (*Hb-18S*) and relative to the expression of the control. A gene with a relative abundance of one is equal to the abundance of 18S rRNA in the same sample in qRT-PCR analysis. The qRT-PCR analyses confirmed the differential expression of the candidate genes as indicated by microarray analysis. The fold change in gene expression (L5M vs OHB) obtained by using microarray experiments compared to the fold change obtained by using qRT-PCR gives a correlation coefficient (R^2^) of 0.84 (Figure 6). The correlation coefficient obtained in our analysis is very good considering that microarray data are semi-quantitative and subject to error for multigene families where different transcripts could hybridize to similar probes on the array. We obtained significantly higher levels of expression of 9 candidate genes by qRT-PCR as compared to microarray analysis (Figure 5). Among 16 candidate genes (9 up-regulated and 7 down-regulated), *Hb-cyn-1 *(GenBank: EX007863) showed highest up-regulation by microarray followed by *Hb-NOSIP *(GenBank: ES411895). Similar results for the other genes are also shown by qRT-PCR, but change in expression level was significantly higher than indicated by microarray. Among seven down regulated genes, qRT-PCR analysis showed significantly higher reduction of five candidate genes as compared to microarray analysis (Figure 5).

## Discussion

Deterioration of traits essential for biological control has been recognized in diverse biological control agents including the insect pathogenic nematode *Hb *[14,15,19]. These traits can deteriorate rapidly and substantially when bio-control agents are isolated from nature and reared in the laboratory, or mass-produced for commercial purposes. Genetic and non-genetic processes may be responsible for trait deterioration in laboratory-cultured bio-control agents and their symbionts. However, the specific mechanisms behind these genetic processes remain unclear. To identify genes associated with trait deterioration in the entomopathogenic nematode *Hb*, we undertook an expression profiling study using custom Roche NimbleGen expression arrays that screened over 15,220 transcripts. To identify the DEGs, expression was compared between two experimental lines of *Hb*; L5M and OHB. Our results showed that trait deterioration of *Hb *induces substantial overall changes in the nematode transcriptome (Figure 1). We observed a few general patterns suggesting that trait deterioration via inbreeding depression, taking place over a short period of time (under 20 passages), can result in massive changes in metabolic processes, cellular transportation and gene translation. In addition, the massive reprogramming of primary and secondary metabolic processes as part of trait-deterioration involved changes in signalling and other regulatory processes. The present study represents the first transcriptional analysis of degradation of beneficial traits in EPNs and highlights several key components of trait deterioration that may be common among biological control agents.

### Experimental design and analysis

The advent of microarrays has enabled the screening of thousands of genes in parallel to assist in candidate gene identification. In this study we used a set of over 15,220 ESTs to construct a cDNA microarray. The main source of ESTs for this array was derived from *Hb *TT01 that interacts symbiotically with *Photorhabdus luminescens *TT01 bacterium. The microarray experiments were conducted in a reference design, where tissue samples from the original parental line, OHB, acted as reference against the inbred line, L5M. The results show 1,185 genes were DE, encompassing diverse functions. We validated our microarray observations by qRT-PCR for several genes that were chosen based on their biological interest as well as spectrum of significance in fold change expression. In general, the results revealed the evolution of altered transcript levels concomitant with trait deterioration, including major changes in the metabolism category, especially in energy, carbohydrate and lipid metabolism.

It is common practice to use an arbitrary transcription differential cut-off (such as twofold) in order to identify changes that may be biologically significant, but our results showed that the majority of DEGs exhibit a small fold change in expression level ranging from 0.38 to 2.45. A number of past studies have shown ineffectiveness of a two-fold cut-off as the basis for filtering out DEGs [24-26] and low-magnitude transcriptional changes have been found to produce functionally significant changes [27-29]. The low amplitude modulation of gene expression (less than two-fold changes) is suggestive of low-magnitude remodelling of the transcriptome, which may be an integral component of an organism's adaptive response to selection on physiological traits. It is possible that the overall evolutionary response of the nematode, even over very small time scales (a few generations) requires coordinated changes among a wide array of genes, and those changes in turn may require reinforcing changes in an even wider array of functionally connected genetic components. We speculate that numerous genes that function in *Hb *are co-ordinately modulated to support the many physiological changes manifested in the evolution of trait deterioration.

Biological gene regulatory networks are highly interconnected systems. Non-linear, synergistic interactions are common. Large number of genes with low-magnitude transcriptional modulation could potentially be just as important in conferring phenotypes and mediating physiological adaptation as the small numbers of genes that show large-magnitude modulations. Our findings suggest that widespread, low-magnitude transcriptional remodelling may be a normal process during physiological adaptation in trait deteriorated nematodes. However, understanding the role of pervasive low-magnitude remodelling may require using computational modelling approaches at a system level, as well as improved technologies for accurately measuring those changes.

### Comparative analysis of differentially expressed genes

We obtained 1,185 genes that were differentially expressed in a deteriorated line of *Hb*. Comparative analysis of these DEGs with those available in various public databases showed that 59% (n = 698) matched *C. elegans *and *C. briggsae *proteins, and 26% (n = 313) matched parasitic nematodes. When these 313 DEGs were compared with a subset of parasitic nematodes, 7% (n = 82) matched animal and human parasitic nematode (AHPN) proteins, suggesting that these genes may participate in parasitism-related activities. Of the remaining DEGs, 19% (n = 231) did not match AHPN sequences that we designated as parasitic nematode specific. A small portion of DEGs 10% (n = 122) did not appear to match any available sequences, indicative of novel *Hb *genes. These findings suggest the potential of discovering new genes and gene functions, genetic networks, and metabolic pathways specific to *Hb *and other EPNs. Similarly, the identification of putatively secreted proteins and expression profiling of the DEGs shared between *Hb *and other parasitic nematodes could be a valuable resource for conducting in-depth research on gene functions that will ultimately elucidate parasitic nematode-specific biological processes.

We found several *Hb *DEGs that are associated with RNA interference (RNAi) phenotypes of *C. elegans*. Our analysis shows differential expression of genes like *egl-8*, *unc-60*, *daf-8*, *daf-21*, *eat-6 *(complete list in Additional file 1), each of which exhibit RNAi phenotypes in *C. elegans*. These genes may prove useful candidates in the ongoing RNAi endeavors for functional genomics studies of EPNs. Interestingly, we found 9 DEGs that matched proteins from various prokaryotic organisms. These transcripts encode ATP synthase (GenBank: FF679373) and a DNA-J class molecular chaperone (GenBank: ES409751). The presence of these transcripts could be the result of horizontal gene transfer (HGT) from bacteria encountered by *Hb *during its life cycle. The presence of sequences of putative prokaryotic origin has already been reported in *Hb *[30] as well as in plant parasitic nematodes [31]. Given the similarity of these sequences to prokaryotic sequences, and presence of poly(A) RNA in the transcript, the possibility that these sequences are bacterial contaminants is low. Our findings of *Hb *DEGs with similarity to prokaryotic sequences identified here do not imply that all these genes have been acquired by HGT, as the null hypothesis remains convergent evolution. However, their presence serves as a first step in identifying a pool of candidates from which parasitism and mutualism-related genes can be explored in the future.

### Functional analysis of differentially expressed genes

Gene Ontology (GO) [22] has been used widely to predict gene function and classification. GO provides a dynamic vocabulary and hierarchy that unifies descriptions of biological, cellular and molecular functions across genomes. We used Blast2GO [23], a sequence-based tool, to assign GO terms, extracting them for each BLAST hit obtained by mapping to extant annotation associations. Though GO analysis showed only one third of the DEGs can be assigned to different functional categories, we observed a clear pattern of changes exhibited by deteriorated nematodes. High numbers of DEGs were assigned to biological processes, including metabolic, developmental, cellular processes and cellular stress. We observed a pattern of changes in primary as well as secondary metabolic processes, indicating that our trait-deteriorated nematodes evolved massive metabolic changes. The molecular function category was dominated by binding, catalytic, transporter, transcriptional regulator and enzyme regulator activities. Such a representation of diverse functional areas is suggestive of coordinated modulation of genes from different functional areas to support the changes undergone during the evolution of trait deterioration.

Biochemical functionality was predicted by mapping all DEGs to pathways using KEGG within Blast2GO. Molecules involved in metabolism (energy, amino acid, carbohydrate and lipid metabolism), neurodegenerative diseases and signal transduction had the highest representation amongst the sequences mapped to KEGG pathways. The enzyme cytochrome c oxidase had the highest mapping to both energy metabolism and neurodegenerative disease categories. Similarly, other enzymes well represented in KEGG pathways are vacuolar ATP synthase (GenBank: EX011485), protein disulfide isomerase (GenBank: EX012905), transglutaminase (Tgase) (GenBank: EX012170), phosphoglycerate dehydrogenase (GenBank: EX013716), NADH dehydrogenase (ubiquinone) (GenBank: ES411557, EX010284), aldehyde dehydrogenase (GenBank: ES411128) and aconitate hydratase (GenBank: EX014674, ES741155). We identified predicted proteins with potential roles in host-parasite interactions, MAPK and T-cell receptor signaling pathway and apoptosis. Although at this stage the precise role of such molecules in the nematode-bacteria-insect host interplay is unclear, they could be involved in manipulating the host's immune response or associated with nematode's innate immune response. Furthermore, we identified families of proteins representing serine, cysteine and metallo-proteinases as well as proteinase inhibitors. While these enzymes are inferred to mediate or modulate proteolytic functions, they may in turn, facilitate the nematode's interaction with its host and symbiont, as the proteinase inhibitors may protect the nematodes against its host's immune system.

### Genes of general and secondary metabolism

Results obtained from our analysis showed that trait deteriorated nematodes undergo massive changes of the transcripts encoding metabolic enzymes and processes. We observed a pattern emerging from our studies suggesting that the trait-deteriorated nematodes down-regulate their primary metabolic processes, which at the same time activate secondary metabolic processes. We also identified significant changes in the dynamics of the genes responsible for energy, amino acid, carbohydrate and lipid metabolism. Enzymes involved in xenobiotic biodegradation, glycan biosynthesis and metabolism and biosynthesis of secondary metabolites were also changed (Additional file 4). These results show that the evolution of trait deterioration can result in metabolic upheavals that could be responsible for reduced pathogenicity. The biggest change was observed in energy metabolism, involving the up-regulation of cytochrome c oxidases (CCO) (GenBank: EX012198) and down-regulation of vacuolar ATPases (V-ATPases) and NADPH-cytochrome P450 (GenBank: ES411356). Cytochrome c oxidase encodes an important enzyme involved in oxidation phosphorylation pathways and thus energy production. In *Cryptococcus neoformans*, the up-regulation of CCOI was shown to be related to stress response of the yeast, which is vital for survival in its hostile host [32]. It is possible that the up-regulation of this mitochondrial gene might be linked to an increased energy production critically important to the survival of *Hb *in a deteriorated condition. The V-ATPases are ATP-dependent proton pumps present in both intracellular and plasma membranes, and function in processes such as receptor recycling, protein processing and degradation [33]. In *C. elegans *H+-V-ATPases are required for development and osmoregulation in animal excretory systems [34] and act as potent lifespan regulators [35]. The down-regulation of V-ATPases is indicative of the deterioration in cellular homeostasis, and general reduction in cellular transportation activities associated with the trait-deteriorated *Hb*.

We observed an interesting transcriptional pattern of genes involved in amino acid, lipid and carbohydrate metabolism, with the majority of the genes being down-regulated. There was up-regulation of sterol metabolism and down-regulation of enzymes in the category of synthases and hydrolases, suggestive of huge shifts in metabolism. Similarly, we observed down-regulation in the category of a dehydrogenase-like aldehyde dehydrogenase, glutamate dehydrogenase, suggesting repression of fermentative pathways. Carbohydrate metabolism was mostly down-regulated with the exception of pyruvate dehydrogenase (GenBank: NP_500340) and phosphoglycolate phosphatase (GenBank: EX910617). Similarly, amino acid metabolism was also mostly down-regulated, except for sorbitol dehydrogenase (SDH) (GenBank: XP_790483), glutathione peroxidase (GenBank: NP_497078) and a few other enzymes (Additional file 4). During anhydrobiosis, nematodes reportedly accumulate polyols like sorbitol and glycerol, which are known to protect animal tissues and cells from injuries caused by freezing or dehydration [36]. As anhydrobiosis is an ametabolic stage, the induction of SDH suggests a general reduction in metabolic activities, and nematodes might be using SDH as a stress survival mechanism. We also observed the differential regulation of enzymes involved in the tricarboxylic acid (TCA) cycle, including the up-regulation of pyruvate dehydrogenase and down-regulation of citrate synthase (GenBank: ES412521), aconitate hydratase (GenBank: ES741155), and dihydrolipoyl dehydrogenase (GenBank: EX010778). Two DEGs encoding fructose-bisphosphate aldolase (FBPA) (GenBank: EG025510, ES744087) were down-regulated in the deteriorated line relative to original line. FBPA is an early step in the glycolysis pathway. The products of this pathway are ATP and pyruvic acid (PVA).

### Potential signal transduction related genes

We identified a set of signal transduction components which likely orchestrate a rapid and general response to a wide range of changes, but also a set of signalling components that may mediate responses more specific to nematode trait deterioration (examples are highlighted in Table 1). Transcriptome patterns associated with signalling during trait deterioration of insect parasitic nematodes have not been well established. We observed differential expression of signalling components like stress-induced-phosphoprotein 1(GenBank: ES740228), phospholipase c beta (GenBank: EX009150), cyclophilin-1 (GenBank: ES411663) and sodium/potassium transporting ATPase (GenBank: ES741918), which are involved generally in stress response and transduction.

The stress-induced-phosphoprotein 1 (*sip-1*) or the Hsc70/Hsp90-organizing protein belongs to a group of co-chaperones, which regulate and assist the major chaperones. *sip-1 *modulates the chaperone activities of linked proteins and also interacts with other chaperones. The loss of *sip-1 *function in *C. elegans *results in embryonic lethality and reduction in life span [37]. Among our DEGs are several transcripts encoding phospholipase c beta of the gene encoded by *C. elegans egl-8*. PLCβ in conjunction *egl-30 *acts in motor neuronswith to directly or indirectly regulate acetylcholine release, thereby modulating locomotion rate and behavior [38]. Also included in our DEGs were two transcripts encoding *C. elegans *cyclophilin (*cyn-1 *and *cyn-5*), a class of peptidyl-prolyl *cis-trans *isomerase (PPIase) enzymes which play an important role in protein folding [39]. These cyclophilins are predicted to be secreted proteins in nematodes that are constitutively expressed [40]. Induced signalling of these genes is likely an attempt on the part of stressed nematodes to combat reduction in life span and maintain the proper functioning of proteins in a changing environment.

A number of transcripts encoding protein tyrosine kinase (GenBank: EX009882) and NADH-cytochrome P450 (GenBank: EX009598) were down-regulated and nitric oxide synthase interacting protein (NOSIP) (GenBank: ES412752) was up-regulated in L5M compared to OHB. Protein tyrosine kinases (PTKs) are important for intra- and inter-cellular communication as well as for survival in eukaryotes and play a major role in signal transduction processes. These proteins are also known to be involved in developmental and differentiation processes of cells [41]. In *C. elegans, a*nother signalling molecule, NADH-cytochrome P450 (NADH-CYP), has been shown to be involved in the detoxification of environmental pollutants and synthesis and degradation of signalling molecules [42]. One of the *C. elegans *CYP isoforms plays an important role in regulating lifecycle progression and the adult life span [43]. The down-regulation of CYP in the deteriorated *Hb *indicates an impaired ability of these nematodes to detoxify environmental pollutants and use of alternative pathways.

NOSIP is an enzyme that regulates nitric oxide (NO) production through binding with high affinity to the carboxyl-terminus of the endothelial nitric oxide synthase (eNOS) oxygenase domain and preventing NO synthesis. NO, a signalling molecule produced by nitric oxide synthase (NOS), is part of the immune response and acts as a neurotransmitter and proliferation signal. It is produced by both parasitic nematodes and their host. The excretory/secretory products from filarial nematodes, which include NO, have been shown to inhibit the proliferation of host cells mediating innate or acquired immunity [44]. Although NO from *Hb *has not been characterized yet, it could play a significant role in invasion and/or suppression of the host immune system. Up-regulation of NOSIP, which negatively regulates NO production, could result in reduced virulence, one of the observed characteristics in the trait-deteriorated *Hb*. In addition to the signalling molecules described here, the discovery of more signalling transcripts from *Hb *adds to the existing knowledge base of dauer-related genes in *C. elegans*, furthering exploration of the importance of signalling components on trait improvement, increased longevity and stress resistance in nematodes.

### Stress and defense related genes

With the advent of microarray technology, researchers can now identify a broad range of genes that are involved in trait deterioration in EPNs. While some genes may be developmental stage-specific, others may be part of a general stress response shared across multiple nematode species. A large portion of the DEGs we found are involved with stress and defense response of nematodes, including two up-regulated transcripts encoding a homolog of *C. elegans hsp-12.6 *(GenBank: EX007554) and *daf-21*(GenBank: EX007741). The *C. elegans daf-21 *gene encodes a member of the HSP90 family of molecular chaperones important for maturation of signal transduction kinases in neurons involved in odorant perception [45]. The post-embryonic phenotype of *Hb *treated with double stranded (ds) *Hba-daf-21 *RNA resulted in abnormal gonad morphology [46]. Another up-regulated gene, *hsp-12.6*, which encodes a small heat shock protein (sHSP) in *C. elegans*, is developmentally regulated but is not up-regulated by a wide range of stressors [47]. Despite its lack of chaperone activity, *hsp-12.6 *regulates the functions of other sHSPs, acting as a co-chaperone with other molecular chaperones [48]. It is possible that *hsp-12.6 *is also developmentally regulated, specifically during the infective stage and plays a significant role in driving the chaperone activities of other Hsps, and conferring protection against oxidative stress. Transcripts encoding homologs of the mitochondrial cytochrome c oxidase (CCO) subunits I, II, and III were abundantly expressed and up-regulated in our trait-deteriorated line. Interestingly, the up-regulated DEGs included 4 transcripts (GenBank: ES740428, ES743969, EX013085, NP_492764) encoding ubiquitin conjugating enzymes (UBCs) and one encoding the 26S proteasome regulatory subunit (GenBank: EX009185). UBCs have been shown to be induced under stress conditions in nematodes [49], and nutrient deprivation in plants has shown to induce ubiquitin degradation of proteins and lipids [50]. It has been suggested that cellular stress results in improperly folded proteins, which are targeted for degradation by ubiquitinization. Interestingly, one of the over-expressed genes in the deteriorated line encoded a chaperonin protein (GenBank: ES743704), which are needed for proper folding of nascent proteins.

Among the down-regulated genes, *eat-6 *encodes an alpha subunit of sodium/potassium ATPase, which in turn affects the NA^+^/K^+^-ATPase activity of membranes. It plays a significant role in the relaxation of pharyngeal muscle, fertility, and also affects body length and life span [51]. Transcriptional profiling showed two transcripts (GenBank: ES744087 and EG025510) encoding fructose-1, 6-bisphosphate (FBP) aldolase, which was down-regulated in the trait-deteriorated nematode line. FBP aldolase, a member of the class I aldolase family, is a glycolytic enzyme that catalyzes the cleavage of FBP into glyceraldehyde 3-phosphate and dihydroxyacetone phosphate [52]. Predicted proteins with potential roles in T-cell receptor (TCR) and transforming growth factor beta (TGF-beta) signaling were also down-regulated. In the parasitic nematode *B. malayi*, a *C. elegans daf-7 *homolog which encodes a member of the TGF-beta superfamily was reported to be involved in manipulation of the host immune response [53]. Although at this stage the precise role of such molecules in host-parasite interactions is not clear, they could be involved in manipulating the host's immune response.

### Dauer and nematode life span regulation

The infective juvenile stage of entomopathogenic nematodes is developmentally similar to the dauer stage in many bacterivorous nematodes, including *C. elegans *and *C. briggsae*. The dauer is a developmentally arrested stage triggered by food deprivation, high population density, and other harsh environmental conditions. Elucidation of this process is of specific interest in the case of entomopathogenic nematodes because the dauer juvenile is the only life stage capable of infecting insects [8]. We observed differential expression of genes which are commonly expressed in dauer and starved adults of *C. elegans*. Transcripts encoding *hsp90 *(GenBank: ES743545), *hsp70 *(GenBank: FF678037) were up-regulated while a GTP-binding ribosomal protein homolog (GenBank: ES410054) and SH3-domain containing protein (GenBank: FF681443) were down-regulated in the trait-deteriorated line compared to original. These genes are more abundantly expressed in dauer than in non-dauer (L3) larvae of *C. elegans *[54]. One of the down-regulated DEGs that we encountered encodes a serine/threonine protein kinase (*unc-51*) (GenBank: FF681332) that is orthologous to *Saccharomyces cerevisiae *autophagy protein. It is required for normal dauer morphogenesis of the *C. elegans daf-2 *mutant [55]. The down-regulation of *unc-51 *may limit reallocation of nutrients in starving cells, such as those in dauer juveniles in free-living nematodes, or infective juveniles in entomopathogenic nematodes. Vacuolar H^+^-ATPases (GenBank: EX011485, NP_508711) were another potent lifespan regulator we found differentially expressed in trait-deteriorated nematodes. These proteins acidify intracellular compartments and act in synaptic transmission and the cell death signaling cascade [56]. Another down-regulated *daf-16 *dependent gene encodes a glucose-6-phosphate isomerase (GenBank: ES740896) homolog which functions in the insulin/IGF-1 pathway to affect lifespan. In mammals, glucose-6-isomerase functions in glycolysis, which influences aging [57]. The DEGs we recovered included 10 transcripts encoding components of the mitochondrial respiratory chain, including ATP synthase and NADH-ubiquinone oxidoreductase (ES411557, EX010284). RNAi of respiratory-chain components decreases body size and slows movement and eating behavior (pumping) of nematodes [58]. Nematodes exposed to stress induce generation of reactive oxygen species (ROS), and therefore it is important for nematodes to have effective ROS scavenging mechanisms. A transcript which encodes a putative Ras related protein (GenBank: ES411895), a *C. elegans rab-33 *homolog, was up-regulated (Figure 5), suggesting that trait-deteriorated nematodes may have to elevate their ROS scavenging mechanisms.

### Refined gene-specific expression using quantitative reverse transcription-PCR

The microarray observations were validated by quantitative reverse transcription-PCR (qRT-PCR) for some representative transcripts (Figure 5). Sixteen genes that were differentially expressed in trait deteriorated nematodes were selected for qRT-PCR validation. For all the selected genes for which primers worked, qRT-PCR validation revealed similar expression kinetics for all the genes tested, indicating reliability of the microarray data (Figure 6). The expression values obtained by qRT-PCR were generally more exaggerated than the corresponding microarray values, as reported in previous studies [59,60]. Although microarray analyses showed low-magnitude change in DEGs (Figure 2), we are able to verify the differential expression by means of qRT-PCR. It is possible that these levels may fall below a technical threshold and therefore do not allow a reliable transcript quantification by using only hybridization-based methods such as microarray analysis. The biological significance of such a change depends on the particular gene under consideration. Therefore, we believe that since the two-fold cut-off is somewhat artificial, its use could lead to misrepresentation of the set of up- or down-regulated genes, resulting in the loss of biologically important information. This conclusion is reinforced by the strong correlation (R^2 ^= 0.84, *P *< 0.05) for all of the transcript-concordant genes that we examined in this study irrespective of their differential regulation.

Microarrays tend to have a low dynamic range, which can lead to small yet significant under-representation of fold change in gene expression [61]. As qRT-PCR has a greater dynamic range, it is often used to validate the observed trends rather than duplicate the fold changes obtained by chip experiments [62-64]. The overall physiological response of an organism or cell to a stimulus may require coordinated changes in a wide array of genes. Those changes in turn may require compensating or reinforcing changes in an even wider array of functionally connected genetic components. Our analysis suggests that low-magnitude expression changes may be of functional significance. It is possible that some genes may show low-magnitude transcriptional modulation but still play a significant role in the resulting physiological response. We speculate that the genes involved in the evolution of EPN trait deterioration are co-ordinately modulated and thus show moderate levels of transcriptional change.

## Conclusions

The present study has given us a first glimpse of the transcriptional analysis of trait deterioration of insect parasitic nematode and represents a starting point for studies in a number of different fundamental and applied areas. In addition to transcriptional profiling using cDNA microarrays, we used comprehensive transcriptional analysis tools for functional annotation at the DNA and protein level. From this study, we identified DEGs which included homologs of *C. elegans *and *C. briggsae*, animal and human parasitic nematodes, prokaryotes, and transcripts specific to parasitic nematodes. These transcripts are particularly interesting, as they may represent genes that are specific to parasitism or to particular EPN species. We also identified a number of potential molecules that are secreted or excreted in the host-parasite interactions, which could serve as a starting point for further experimental analyses. The secreted proteins that lacked homology with other free-living and animal parasitic nematode proteins could be involved in *Hb*-*P. luminescens *symbiosis-specific processes, or play vital roles in insect parasitism and suppression of host defense mechanisms. The comparison of DEGs with *C. elegans*, *C. briggsae *and other nematodes revealed common, but also parasitic nematode-specific genes. As the most closely related major entomopathogen to *C. elegans*, *Hb *provides an attractive near-term application for using a model organism to better understand the origin and evolution of interspecies interactions (e.g. parasitism, mutualism and vector-borne disease) and to enhance our understanding of the mechanisms underlying trait deterioration in biological control agents. Beyond functional analysis of *Hb *genes, clear research avenues are available to apply this information to improve the beneficial traits of bio-control agents and better understand the fundamental aspects of nematode parasitism and mutualism.

## Methods

### Nematode culture

A deteriorated population of *Hb *was created by sub-culturing different experimental lines of nematode-bacterium complex over 20 passages in larvae of the greater wax moth, *Galleria mellonella *[14]. The original parental strain (OHB) was maintained in Ringer's solution without sub-culturing while the inbred line (L5M) was continuously cultured in *G. mellonella*. Both OHB and L5M were cultured identically in *G. mellonella *larvae [65] and emerging infective juveniles (IJs) were collected using White traps [66]. The IJs of both lines were stored in Ringer's solution at 16°C for not more than one day before used for RNA extraction.

### RNA extraction and cDNA synthesis

Total RNA was isolated from four biological replicates of IJs of each strain. The IJs (~8-10,000) were transferred to 10 volumes of Trizol Reagent (Molecular Research Center Inc., Cincinnati, OH) and exposed to freeze thaw cycles using liquid nitrogen and 37°C water bath. The suspension was ground using mortar and pestle and vortexed. RNA was phase separated using chloroform, precipitated by isopropanol and pelleted. At least three sub-samples from each biological replicates were used for RNA extraction and total RNA was pooled. Total RNA was converted to double stranded cDNA using SuperScript™ double-stranded cDNA synthesis kit (Invitrogen Corporation, Carlsbad, CA). Double-stranded cDNA was quantified and quality checked by using Agilent 2100 Bioanalyzer (Agilent Technologies, Inc., Santa Clara, CA).

### Array design and data analysis

Microarrays containing probes against 15,220 *Hb *ESTs assembled from ESTs under GenBank accession numbers [GenBank:EG025323] - [GenBank:EG025806], [GenBank:ES408468] - [GenBank:ES414355], [GenBank:ES738967] - [GenBank:ES744677], [GenBank:EX006911] - [GenBank:EX015306], [GenBank:EX910019] - [GenBank:EX916843], and [GenBank:FF678120] - [GenBank:FF681586] were designed and manufactured by Roche NimbleGen (Roche NimbleGen Inc.). Double-stranded cDNA extracted from four biological replicates of each strain of *Hb *were shipped to Roche NimbleGen for labelling, hybridization, data collection and normalization according to established manufacturer's protocols. Briefly, single color (Cy3) fluorescently labelled cDNA samples were hybridized to the arrays, and signal intensities were obtained on a microarray scanner. Data from all eight arrays were then normalized using the Robust Multichip Average (RMA) algorithm [67]. A two-group (inbred vs. parental strain) statistical analysis using a two-tailed Student's t-test was performed to identify differentially expressed genes. The complete set of microarray data is accessible through the Gene Expression Omnibus at the National Center for Biotechnology Information (NCBI) under accession number GSE19152.

### Sequence analysis

The differentially expressed (DE) EST sequences representing contamination from bacterial, yeast or fungal sources were identified using the BLASTN algorithm [68] and removed from further analyses. ESTs were compared to the sequences in GenBank's non-redundant (nr) and Uniprot database using tBLASTX and BLASTX [69] algorithms, and *C. elegans *WS200 (11th release of WormBase [20]) database using the BLASTX algorithm [68,70]. ESTs were also compared to the available animal and human parasitic nematodes (AHPNs) and plant parasitic nematode (PPNs) ESTs using tBLASTX. The DE ESTs with no significant matches to proteins of AHPNs and PPNs but matched to *Hb *and other EPNs were designated as parasitic nematode-specific ESTs, which were further characterized.

In order to minimize the number of false positive predictions from the peptides inferred from DE ESTs, secreted proteins were predicted using a combination of two programs. Firstly, SignalP 3.0 [70] was used to predict the presence of secretory signal peptides (SPs) for each predicted DE EST proteins. A signal sequence was considered present when it was predicted both by the artificial neural network and the hidden Markov model prediction approaches (SignalPNN and SignalP-HMM). In order to exclude the erroneous prediction of putative Transmembrane (TM) sequences as signal sequences, TMHMM [71], a membrane topology prediction program, was then applied. Identification of sequence similarity was performed using BLAST analyses against nr (non-redundant) databases.

### Functional analysis and pathway assignment

Gene ontology (GO) term annotation and function-based analysis of DEGs were performed using Blast2GO (V 1.6.2) [23]. GO terms for each of the three main categories (biological process, molecular function, and cellular component) was obtained from sequence similarity using the application default parameters. From these annotations, pie charts were made using 2nd level GO terms based on biological process, molecular function, and cellular component. Pathway assignments were carried out according to Kyoto encyclopedia of genes and genomes (KEGG) [21] mapping. Enzyme commission (EC) [72] numbers were assigned to DE sequences that had BLASTX scores with a cut-off value of E = 10^-5 ^or less upon searching protein databases. The sequences were mapped to KEGG biochemical pathways according to the EC distribution in the pathway database.

### Primer design

A set of DEGs from different functional areas were selected and gene-specific primers were designed. All the primers used in quantitative real-time PCR (qRT-PCR) were designed using IDT SciTools (Integrated DNA Technologies, Coralville, IA) by aligning EST sequences with similar sequences from NCBI and synthesized by Operon (Operon Biotechnologies Inc., Huntsville, AL). The sequences of the primers and product sizes are listed in Additional file 5.

### Validation of differential expression by quantitative reverse transcription-PCR

Total RNA extracted from L5M and OHB nematodes was reverse transcribed using ImProm-II™ reverse transcriptase (Promega corporation, Madison, WI) and subjected to qRT-PCR analysis using LightCycler 480 SYBER Green I mastermix and gene-specific primers in a LightCycler 480 RT-PCR system (Roche Applied Science, Mannheim, Germany) equipped with LightCycler 480 software. High-resolution gel electrophoresis was used to verify that the qRT-PCR amplification product from each examined gene was a single-band product. Thermal cycling was performed in accordance with the manufacturer's instructions for a total of 45 cycles at an annealing temperature of 58°C for each primer pair. Quantitative RT-PCR analysis was performed with LightCycler 480 software, the threshold cycle was automatically calculated by the second-derivative maximum method.

### Data analysis

In qRT-PCR experiments, changes in target gene expression were calculated using equation 2^-ΔΔCT ^[73]. The fold change in the target gene, normalized to 18S rRNA (*Hb-18s*) and relative to the expression of control, was calculated for each sample. A gene with a relative abundance of one is equal to the abundance of 18S rRNA in the same sample. An F-test at a significance level of *P *< 0.05 was used to compare the ratio of the mean gene expression of L5M samples with that of OHB. To minimize mRNA quantification errors, genomic DNA contamination biases and to correct for inter-sample variations, we used 18s ribosomal RNAs (rRNAs) of *Hb *as an internal control. The correlation coefficient (R^2^) between qRT-PCR and microarray data was calculated using NCSS [74]. For microarray experiments, gene expression above and below one (1.0) was considered as up- and down-regulation for further analysis. Significant differential expression between two lines was calculated using the student t-test (*P *< 0.05).

## Authors' contributions

BNA carried out most of the work described here including conception of experiments, analysis and interpretation of data, functional characterization and validation of differentially expressed genes and drafting the manuscript. CYL contributed to conception and design of microarray experiments, acquisition of data and statistical analyses. XB, TAC, PSG, PWS, DIS and BJA contributed to EST sequencing, sequence annotation and assembly. ARD, JMC, DIS, ALB, RG and BJA performed breeding experiments and stress tolerance, fecundity and pathogenicity assays. BJA contributed to conception and design of experiments, supervision of the work and critical review of the manuscript. All authors critically reviewed and approved the final manuscript.

## Supplementary Material

Additional file 1 **Differentially expressed genes in trait deteriorated *Heterorhabditis bacteriophora *exhibiting RNAi phenotype similar to *Caenorhabditis elegans***. The RNAi phenotypes were identified by comparison of differentially expressed ESTs with *Caenorhabditis elegans *database (WS200). The table also provides corresponding RNAi phenotypes in *C. elegans *and their annotations. Click here for file

Additional file 2 **Secreted proteins predicted from differentially expressed ESTs from trait deteriorated *Heterorhabditis bacteriophora***. Signal sequence was considered present when predicted both by SignalPNN and SignalP-HMM [68]. Putative transmembrane (TM) sequences were excluded by applying a topology prediction program TMHMM [69]. Click here for file

Additional file 3 **KEGG biochemical mappings for *Heterorhabditis bacteriophora *differentially expressed ESTs**. Differentially expressed ESTs were mapped to different biochemical pathways via Kyoto encyclopedia of genes and genomes (KEGG) [24]. Click here for file

Additional file 4 **Metabolism related genes exhibiting differential expression between the trait-deteriorated, inbred line (L5M) and its original parental line (OHB) in *Heterorhabditis bacteriophora***. The table provides the most represented metabolism related genes which were differentially expressed in the deteriorated line as compared to original line of *Heterorhabditis bacteriophora*. These genes were mapped to different biochemical pathways via Kyoto encyclopedia of genes and genomes (KEGG) [24]. Click here for file

Additional file 5 **Gene-specific primer sequences used for quantitative reverse transcription-PCR analysis**. Primers were designed by aligning the EST sequences with their putative homologue from GenBank. Click here for file
