# Supplementary material for: Transcriptional profiling of trait deterioration in the insect pathogenic nematode Heterorhabditis bacteriophora
Source: BMC Genomics. 2009 Dec 15;10:609. doi: 10.1186/1471-2164-10-609 (PMC2805696; doi:10.1186/1471-2164-10-609)
Supplement: Additional file 1 — Differentially expressed genes in trait deteriorated Heterorhabditis bacteriophora exhibiting RNAi phenotype similar to Caenorhabditis elegans. The RNAi phenotypes were identified by comparison of differentially expressed ESTs with Caenorhabditis elegans database (WS200). The table also provides corresponding RNAi phenotypes in C. elegans and their annotations. [file 1471-2164-10-609-S1.PDF]

Additional file 1- Differentially expressed genes in trait deteriorated *Heterorhabditis bacteriophora* exhibiting RNAi phenotype similar to *Caenorhabditis elegans*.

**Accession number**	***C. elegans* homolog**	**Annotation**	**E-value**	**Fold change (P value)**	**RNAi phenotype[£]**
EX009150	WBGene00001177 (egl-80	Phospholipase C beta	1e-38	0.89 (0.004)	Egg laying defective
EX915077	WBGene00006794 (unc-60)	Actin depolymerizing factor 1	2e-66	0.87 (0.005)	Uncoordinated
ES411502	WBGene00006810 (unc-78)	Actin-interacting protein 1	1e-42	0.81 (0.006)	Uncoordinated
ES744585	WBGene00006810 (unc-78)	Actin-interacting protein 1	4e-47	0.85 (0.048)	Uncoordinated
FF681332	WBGene00006786 (unc-51)	Serine/threonine protein kinase	5e-63	0.80 (0.007)	Uncoordinated
EX007741	WBGene00000915 (daf-21)	Heat shock protein 90	5e-63	1.14 (0.024)	Abnormal dauer formation
ES743277	WBGene00006801 (unc-68)	Ryanodine receptor family	1e-90	0.75 (0.033)	Uncoordinated
ES741918	WBGene00001137 (eat-6)	Sodium/potassium ATPase	3e-94	0.63 (0.039)	Abnormal pharyngeal pumping
ES743931	WBGene00006789 (unc-54)	Myosin class II heavy chain	1e-104	0.73 (0.041)	Uncoordinated
ES741407	WBGene00006754 (unc-15)	Paramyosin	5e-77	0.69 (0.044)	Uncoordinated
ES412571	WBGene00005026 (sqv-8)	Glucuronosyl transferase	1e-72	1.15 (0.044)	Squashed vulva
EX010968	WBGene00019322 (dpy-14)	S-adenosylhomocysteine hydrolase	1e-101	0.79 (0.047)	Dumpy
NP_492321*	WBGene00000904 (daf-8)	SMAD protein	5e-52	1.18 (0.007)	Abnormal dauer formation
NP_001021093*	WBGene00006819 (unc-87)	C-terminal repeat of calponin	1e-174	1.12 (0.020)	Uncoordinated

*Homolog accession number.

[£]Phenotype according to WormBase database.
