# Supplementary material for: Transcriptional profiling of trait deterioration in the insect pathogenic nematode Heterorhabditis bacteriophora
Source: BMC Genomics. 2009 Dec 15;10:609. doi: 10.1186/1471-2164-10-609 (PMC2805696; doi:10.1186/1471-2164-10-609)
Supplement: Additional file 2 — Secreted proteins predicted from differentially expressed ESTs from trait deteriorated Heterorhabditis bacteriophora. Signal sequence was considered present when predicted both by SignalPNN and SignalP-HMM [68]. Putative transmembrane (TM) sequences were excluded by applying a topology prediction program TMHMM [69]. [file 1471-2164-10-609-S2.PDF]

Additional file 2- Secreted proteins predicted from differentially expressed ESTs from trait deteriorated *Heterorhabditis bacteriophora*.

Number	Sequence ID	Residue	SP	Description (top NR hit)	E-value	% Identity (aa)	RNAi phenotype in *C. elegans*
1	149396870	322	18	Hypothetical protein CBG20736 (*Caenorhabditis elegans*)	7e-87	162/201 (80%)	None
2	156785065	266	18	40S ribosomal protein S13 (*Salmo salar*)	3e-15	15/20 (75%)	-
3	149399353	231	19	Cytochrome c oxidase subunit II (*Heterorhabditis bacteriophora*)	1e-27	62/68 (92%)	-
4	145974548	593	18	Hypothetical protein CBG20880 (*Caenorhabditis briggsae* AF16)	2e-08	62/192 (32%)	-
5	183184914	607	15	Serine/threonine-protein kinase (*unc-51*) (*Caenorhabditis elegans*)	2e-104	182/220 (82%)	dumpy (Dpy), paralyzed (Prl), egg laying defective (Egl_D), locomotion abnormal (Unc)
6	145970758	173	15	Hyptothetical protein CBG14277 (*Caenorhabditis briggsae* AF16)	2e-19	57/173 (32%)	expulsion defective (aex-3)
7	156781521	163	15	Hypothetical protein KO7B1.6 (*Caenorhabditis elegans*)	1e-14	57/121 (47%)	-
8	156785987	169	18	RIKEN cDNA 2610029G23 (*Brugia malayi*)	3e-26	55/107 (76%)	-
9	145974570	62	26	None	None	None	None
10	156786049	183	19	Tropomyosin family protein (*Brugia malayi*)	3e-25	59/73 (80%)	-
11	158948474	325	18	Carbonic anhydrase isoform III (*Ostertagia ostertagi*)	4e-97	167/207 (80%)	None
12	145972366	88	19	60S ribosomal protein L31 (*Salmo salar*)	2e-34	15/16 (93%)	maternal sterile (Ste), embryonic lethal (Emb), lethal (Let), molt defect (Mlt), larval lethal (Lvl), protruding vulva (Pvl)
13	156784162	420	19	Proteasome regulatory particle, non-ATPase-like (*Caenorhabditis elegans*)	1e-49	113/150 (75%)	embryonic lethal (Emb), maternal sterile (Ste), sick (Sck), lethal (Let), transgene expression increased
14	183184800	231	20	Cytochrome c oxidase subunit II (*Heterorhabditis bacteriophora*)	4e-13	35/42 (83%)	embryonic lethal (Emb), extended life span (Age), larval arrest (Lva), sterile progeny (Stp)

15	149396570	127	18	Hypothetical protein CBG20110 (*Caenorhabditis briggsae* AF16)	5e-28	62/111 (55%)	-
16	158952769	339	19	Hypothetical protein ZC477.5 (*Caenorhabditis elegans*)	3e-10	53/201 (26%)	None
17	145971764	540	18	Hypothetical protein F56F10.1 (*Caenorhabditis elegans*)	3e-59	127/209 (60%)	None
18	156782545	216	19	Thioredoxin-like protein p19 precurson (*Brugia malayi*)	1e-56	128/152 (84%)	None
19	145974548	151	19	Hypothetical protein CBG20880 (*Caenorhabditis briggsae* AF16)	2e-08	62/192 (32%)	-
20	145971378	82	15	T Cell Lineage defect family member (*tcl-2*) (*Caenorhabditis elegans*)	2e-16	19/49 (38%)	protruding vulva (Pvl)
21	145971524	88	19	None	None	None	None
22	145973325	395	19	Phosphoglucomutase/phosphomanno-mutase (*Brugia malayi*)	1e-36	86/173 (49%)	-
23	156784130	91	25	U6 snRNA-associated Sm-like protein LSm5 (*Brugia malayi*)	1e-33	71/74 (95%)	-
24	145974038	285	18	U2AF splicing factor family member (*uaf-2*) (*Caenorhabditis elegans*)	9e-73	127/141 (90%)	maternal sterile (Ste), embryonic lethal (Emb), protruding vulva (Pvl), locomotion abnormal (Unc), molt defect (Mlt), clear (Clr)
25	156783078	709	19	High temperature-induced Dauer formation family member (*hid-1*) (*Caenorhabditis elegans*)	2e-104	182/220 (82%)	Maternal sterile (Ste), slow growth (Gro)
26	156779371	152	16	Arp2/3 complex component family member (*arx-7*) (*Caenorhabditis elegans*)	3e-52	100/152 (65%)	larval arrest (Lva), sick (Sck), lethal (Let), sterile progeny (Stp), locomotion abnormal (Unc), sterile (Ste), embryonic lethal (Emb)
27	183182159	61	25	None	None	None	None
28	149397618	93	18	Ferritin, middle subunit (*Salmo salar*)	1e-22	18/29 (62%)	-
29	183185634	107	20	None	None	None	-
30	149396192	248	18	Histidyl tRNA synthetase (*hrs-1*) (*Caenorhabditis elegans*)	6e-36	47/52 (90%)	embryonic lethal (Emb), slow growth (Gro), reduced brood size, larval arrest (Lva), sterile progeny (Stp), small
31	149396972	543	27	Chaperonin containing TCP-1 family member (*cct-3*) (*Caenorhabditis elegans*)	8e-75	138/181 (76%)	larval arrest (Lva), lethal (Let), sterile progeny (Stp), embryonic lethal (Emb), microtubule

							polymerization abnormal
32	149399615	606	18	Hypothetical protein CB04632 (*Caenorhabditis briggsae* AF16)	5e-106	188/196 (95%)	-
33	183184503	114	16	None	None	None	None
34	183185079	214	21	G protein-coupled receptor 89 (*Apis melifera*)	7e-22	39/44 (97%)	-
35	156779517	243	18	Hypothetical protein CBG13057 (*Caenorhabditis briggsae* AF16)	2e-45	110/201 (54%)	None
36	145970704	318	19	Hypothetical protein BRAFLDRAFT_113873 (*Branchiostoma floridae*)	2e-19	48/122 (39%)	-
37	145970758	184	15	Synaptosomal-associated protein 25 (*Oikopleura dioica*)	3e-12	43/143 (30%)	-
38	158951523	150	27	Hypothetical protein CBG00524 (*Caenorhabditis briggsae* AF16)	3e-08	30/36 (83%)	None
39	149400747	452	18	Hypothetical protein M60.7 (*Caenorhabditis elegans*)	2e-77	142/205 (69%)	None
40	156783801	159	18	Adenosine deaminases acting on RNA (*adr-1*) (*Caenorhabditis elegans*)	2e-09	48/142 (47%)	larval arrest (Lva), reduced brood size, molt defect (Mlt)
41	145972572	513	19	Amino acid transporter family member (*aat-6*) (*Caenorhabditis elegans*)	8e-59	111/149 (74%)	None
42	156780887	387	19	Hypothetical protein F13H8.7 (*Caenorhabditis elegans*)	1e-66	130/191 (68%)	maternal sterile (Ste)
43	156779893	418	18	Dehydrogenases, short chain family member (*dhs-6)* (*Caenorhabditis elegans*)	1e-94	169/198 (85%)	None
44	156780513	156	19	Hypothetical protein F44E2.3 (*Caenorhabditis elegans*)	6e-09	45/81 (55%)	None
45	156779401	82	19	60S ribosomal protein L10 (*Salmo salar*)	1e-21	17/23 (73%)	-
46	145974364	537	15	Hypothetical protein (*Caenorhabditis briggsae* AF16)	5e-90	176/191 (92%)	-
47	183184910	474	16	Hypothetical protein ZC434.3 (*Caenorhabditis elegans*)	7e-46	87/95 (91%)	None
48	183182897	504	17	Hypothetical protein T20B12.3 (*Caenorhabditis elegans*)	5e-29	71/127 (55%)	maternal sterile (Ste), slow growth (Gro), larval arrest (Lva), early larval lethal (Lvl),

							embryonic lethal (Emb), locomotion abnormal (Unc), nicotine hypersensitive, sterile (Ste)
49	183182197	133	16	None	None	None	None
50	149399416	196	18	Calumenin (calcium-binding protein) homolog family member (*calu-1*) (*Caenorhabditis elegans*)	1e-13	37/41 (90%)	shortened life span (Age), locomotion abnormal (Unc), lethal (Let), molt defect (Mlt), larval arrest (Lva), clear (Clr), Sterile (Ste), larval lethal (Lvl)
51	158947668	131	17	Transthyretin-related family domain family member (*ttr-17*) (*Caenorhabditis elegans*)	1e-41	81/125 (64%)	None
52	156784100	216	15	Thioredoxin-like protein p19 precursor (*Brugia malayi*)	5e-60	134/160 (83%)	-
53	156779174	463	18	Hypothetical protein CBG16828 (*Caenorhabditis briggsae* AF16)	9e-105	188/190 (98%)	None
54	158953609	211	28	None	None	None	-
55	183183646	81	24	None	None	None	-
56	158953311	305	18	Synthetic lethal with Mec family member (*sym-3*) (*Caenorhabditis elegans*)	3e-61	125/206 (60%)	None
57	183183887	680	16	Hypothetical protein NELLACOT_O1747 (*Neisseria lactamica*)	1e-19	21/54 (38%)	-
58	149400942	333	18	Hypothetical protein (*Brugia malayi*)	2e-29	82/188 (43%)	-
59	145972366	88	18	60S ribosomal protein L31 (*Salmo salar*)	1e-34	15/16 (93%)	-
60	156783907	606	15	Hypothetical protein CBG04632 (*Caenorhabditis briggsae* AF16)	3e-105	172/180 (95%)	None
61	145974496	188	15	Hypothetical protein CBG22129 (*Caenorhabditis briggsae* AF16)	7e-47	93/124 (75%)	None
62	156780714	477	18	K-Voltage-gated sensory channel family member (*kvs-1*) (*Caenorhabditis elegans*)	5e-42	88/167 (52%)	None
63	156783198	373	17	Hypothetical protein F15B9.10 (*Caenorhabditis elegans*)	2e-80	142/177 (80%)	fat content increased
64	149400838	523	19	Aldehyde dehydrogenase family member (*alh-8*) (*Caenorhabditis elegans*)	1e-75	149/182 (81%)	None
65	149398966	288	18	Hsp70-interacting protein, putative (*Aedes aegypti*)	1e-34	79/171 (46%)	-

66	145974137	309	20	Hypothetical protein Y39F10A.3 (*Caenorhabditis elegans*)	5e-38	78/180 (43%)	None
67	145969064	172	24	None	None	None	None
68	156784479	222	18	60S ribosomal protein L18a (*Salmo salar*)	4e-06	23/24 (95%)	-
69	183183626	341	16	Cysteine proteinase (*Ancylostoma ceylanicum*)	3e-11	40/98 (40%)	-
70	156785471	948	15	CBR-UNC-52 (*Caenorhabditis briggsae*)	9e-101	170/221 (76%)	None
71	156781116	398	18	Aspartyl protease family member (asp-3) (*Caenorhabditis elegans*)	3e-71	128/212 (60%)	cell death abnormal (Ced), life span abnormal (Age)
72	149398893	101	18	Hypothetical protein F32D1.9 (*Caenorhabditis elegans*)	1e-15	25/66 (37%)	
73	183182275	110	15	None	None	None	None
74	156779387	155	18	60S ribosomal protein L36 (*Salmo salar*)	1e-27	14/15 (93%)	embryonic lethal (Emb)
75	149398775	248	19	Mitochondrial 2-oxoglutarate/malate carrier protein (*Salmo salar*)	8e-16	51/161 (31%)	-
76	149396248	484	18	Putative protein disulfide isomerase 1 (*Dictyocaulus viviparous*)	4e-98	168/211 (79%)	-
77	183184854	150	18	None	None	None	None
78	156786713	286	18	None	None	None	-
79	156786506	216	19	3’-5’ exoribonuclease CSL4 homolog (*Brugia malayi*)	4e-54	106/207 (51%)	-
80	183182980	208	22	Hypothetical protein CBG16402 (*Caenorhabditis briggsae* AF16)	5e-15	49/105 (46%)	None
81	156779132	192	18	Cyclophilin family member (*cyn-1*) (*Caenorhabditis elegans*)	3e-66	131/178 (73%)	None
82	156779631	231	24	Cytochrome c oxidase subunit II (*Heterorhabditis bacteriophora*)	4e-53	138/174 (79%)	-
83	contig_1263	282	15	Diadenosine tetraphosphatase (*Burkholderia mallei*)	1e-37	24/98 (24%)	-
84	contig_1631	530	21	Hypothetical protein CBG11507 (*Caenorhabditis briggsae* AF16)	5e-77	165/303 (54%)	None

85	contig_1678	436	17	Hypothetical protein CBG19706 (*Caenorhabditis briggsae* AF16)	2e-41	79/123 (64%)	None
86	contig_2035	443	18	Similar to KIAA1379 protein (*Monodelphis domestica*)	2e-42	104/235 (44%)	-
87	contig_2070	352	16	Hypothetical protein CBG21051 (*Caenorhabditis briggsae* AF16)	2e-38	78/98 (79%)	None
88	contig_230	255	24	Hypothetical protein CBG03983 (*Caenorhabditis briggsae* AF16)	5e-103	192/255 (75%)	None
89	contig_2418	269	15	Protein K10D6.2b (*Caenorhabditis elegans*)	2e-65	120/170 (70%)	-
90	contig_2448	217	16	SJCHGC04950 protein (*Schistosoma japonicum*)	1e-22	36/141 (25%)	-
91	contig_2455	373	18	Hypothetical protein CBG09043 (*Caenorhabditis briggsae* AF16)	7e-21	52/117 (44%)	None
92	contig_2463	89	31	None	None	None	None
93	contig_2547	296	21	Hypothetical protein (*Caenorhabditis elegans*)	1e-42	19/27 (70%)	-
94	contig_259	152	23	Hypothetical protein CO6A5.1 (*Caenorhabditis elegans*)	1e-08	33/135 (24%)	None
95	contig_2597	332	19	Similar to Not3 (*Ciona intestinalis*)	1e-11	16/34 (47%)	-
96	contig_2633	165	16	Hypothetical protein F23H12.3 (*Caenorhabditis elegans*)	5e-54	104/142 (73%)	-
97	contig_3009	569	15	Hypothetical protein (*Paramecium tetraurelia*)	1e-13	35/157 (22%)	-
98	contig_304	612	16	Hypothetical protein CBG02033 (*Caenorhabditis briggsae* AF16)	1e-10	31/55 (56%)	None
99	contig_546	382	18	Ce protein C41C4.6 confirmed by transcript (*ulp-4*) (*Caenorhabditis elegans*)	8e-64	113/174 (64%)	aldicarb resistant (Ric), lethal (Let), embryonic lethal (Emb), patchy coloration (Pch), egg laying abnormal (Egl), dumpy (Dpy), sterile (Ste)
100	contig_860	56	20	None	None	None	None
101	contig_864	585	15	Hypothetical protein CBG14624 (*Caenorhabditis briggsae* AF16)	2e-158	208/310 (67%)	None
