# Supplementary material for: Transcriptional profiling of trait deterioration in the insect pathogenic nematode Heterorhabditis bacteriophora
Source: BMC Genomics. 2009 Dec 15;10:609. doi: 10.1186/1471-2164-10-609 (PMC2805696; doi:10.1186/1471-2164-10-609)
Supplement: Additional file 4 — Metabolism related genes exhibiting differential expression between the trait-deteriorated, inbred line (L5M) and its original parental line (OHB) in Heterorhabditis bacteriophora. The table provides the most represented metabolism related genes which were differentially expressed in the deteriorated line as compared to original line of Heterorhabditis bacteriophora. These genes were mapped to different biochemical pathways via Kyoto encyclopedia of genes and genomes (KEGG) [24]. [file 1471-2164-10-609-S4.PDF]

Additional file 4-Metabolism related genes exhibiting differential expression between inbred (L5M) and original parental line (OHB) in *Heterorhabditis bacteriophora*.

Sequence ID	GenBank accession number	Annotation	Fold change	P- value[1]
**Energy metabolism (56)**				
183183314	FF679373	ATP synthase beta subunit	0.91	0.004
156783580	EX011485	Vacuolar ATP synthase subunit H 2	0.65	0.013
149396932	ES740341	H+-transporting two-sector ATPase	0.73	0.013
145973706	ES413394	H+-transporting two-sector ATPase	0.77	0.020
149399615	ES743024	Vacuolar ATP synthase subunit H 2	0.66	0.022
115342469	EG025722	H+-transporting two-sector ATPase	0.88	0.023
149400813	ES744222	Vacuolar ATPase subunit H 2	0.84	0.027
145974364	ES414052	H+-transporting two-sector ATPase	0.85	0.031
156783907	EX011812	Vacuolar ATPase subunit H 2	0.64	0.034
156783658	EX011563	H+-transporting two-sector ATPase	0.76	0.047
contig_1679	NP_508711*	Vacuolar H ATPase family member	1.10	0.011
158948474	EX910693	Carbonate dehydratase	1.07	0.012
156786782	EX014687	Glutamate dehydrogenase	0.80	0.009
156784293	EX012198	Cytochrome c oxidase II	1.52	0.001
156779958	EX007863	Cytochrome c oxidase II	1.58	0.002
158949666	EX911878	Cytochrome c oxidase II	1.48	0.004
145972807	ES412495	Cytochrome c oxidase I	1.63	0.004
156783031	EX010936	Cytochrome c oxidase III	1.37	0.004
183184677	FF679196	Cytochrome c oxidase II	1.44	0.005
149396629	ES740038	Cytochrome c oxidase I	1.62	0.006
156781873	EX009778	Cytochrome c oxidase I	1.49	0.006
156783305	EX011210	Cytochrome c oxidase II	1.56	0.006
149400917	ES744326	Cytochrome c oxidase I	1.47	0.006
156780157	EX008062	Cytochrome c oxidase I	1.63	0.007
156779528	EX007433	Cytochrome c oxidase II	1.61	0.008
145974553	ES414241	Cytochrome c oxidase II	1.36	0.008
156782059	EX009964	Cytochrome c oxidase II	1.35	0.008
156786865	EX014770	Cytochrome c oxidase II	1.52	0.009
149397515	ES740924	Cytochrome c oxidase II	1.48	0.009
145972076	ES411764	Cytochrome c oxidase II	1.57	0.011
156784525	EX012430	Cytochrome c oxidase II	1.53	0.011
156786949	EX014854	Cytochrome c oxidase II	1.54	0.012
149400317	ES743726	Cytochrome c oxidase II	1.47	0.012
156784522	EX012427	Cytochrome c oxidase II	1.57	0.012
145971774	ES411462	Cytochrome c oxidase I	1.51	0.012
149398997	ES742406	Cytochrome c oxidase II	1.45	0.013
183184800	FF681870	Cytochrome c oxidase II	1.20	0.013
158948829	EX910557	Cytochrome c oxidase III	1.60	0.013
115342293	EG025546	Cytochrome c oxidase I	1.54	0.014
156781873	EX009778	Cytochrome c oxidase I	1.49	0.006
156780089	EX007994	Cytochrome c oxidase II	1.46	0.015
158951243	EX913568	Cytochrome c oxidase III	1.26	0.015
149399597	ES743006	Cytochrome c oxidase III	1.52	0.016
156786450	EX014355	Cytochrome c oxidase III	1.60	0.018
156783557	EX011462	Cytochrome c oxidase II	1.39	0.020
156786456	EX014361	Cytochrome c oxidase III	1.55	0.022
115342293	EG025546	Cytochrome c oxidase I	1.45	0.022
156785587	EX013492	Cytochrome c oxidase II	1.48	0.026
156786492	EX014397	Cytochrome c oxidase II	1.43	0.028

156787076	EX014981	Cytochrome c oxidase III	1.46	0.029
158951856	EX914214	Cytochrome c oxidase II	1.48	0.029
145970526	ES410214	Cytochrome c oxidase I	1.49	0.031
156779126	EX007031	Cytochrome c oxidase II	1.46	0.043
156782590	EX010495	Cytochrome c oxidase I	1.35	0.043
156779631	EX007536	Cytochrome c oxidase II	1.26	0.046
156786480	EX014385	Cytochrome c oxidase VIb	0.85	0.049
**Amino acid metabolism (29)**				
149397203	ES740612	Aspartate-semialdehyde dehydrogenase	0.47	0.043
149396870	ES740279	Phosphoglycerate dehydrogenase	0.70	0.001
156784717	EX012622	Phosphoglycerate dehydrogenase	0.69	0.002
149398107	ES741516	Phosphoglycerate dehydrogenase	0.75	0.005
149400313	ES743722	Phosphoglycerate dehydrogenase	0.68	0.005
158948008	EX910309	Phosphoglycerate dehydrogenase	0.74	0.009
156781460	EX009365	Phosphoglycerate dehydrogenase	0.80	0.023
149397957	ES741366	Serine-tRNA ligase	0.75	0.038
145973942	ES413630	Betaine-aldehyde dehydrogenase	0.88	0.005
149397016	ES740425	Aminomethyltransferase	0.81	0.020
149397016	ES740425	Sarcosine oxidase	1.22	0.041
145970789	ES410477	Threonine-tRNA ligase	1.09	0.037
156782597	EX010502	Phosphoserine phosphatase	0.81	0.032
156782873	EX010778	Dihydrolipoyl dehydrogenase	0.77	0.047
145973064	ES412752	Nitric-oxide synthase interacting protein	1.25	0.019
156780809	EX008714	Proline dioxygenase	0.65	0.044
149400835	ES744244	Ornithine aminotransferase	1.28	0.046
156786720	EX014625	3-hydroxyisobutyrate dehydrogenase	1 .17	0.003
149400838	ES744247	1-pyrroline-5-carboxylate dehydrogenase	0.86	0.037
156784265	EX012170	Glutamate-ammonia ligase	0.60	0.046
183185277	FF680848	Glutamate dehydrogenase [NAD(P)+]	0.85	0.035
145970682	ES410370	4-hydroxyphenylpyruvate dioxygenase	0.46	0.009
149396404	ES739813	4-hydroxyphenylpyruvate dioxygenase	0.48	0.042
145973110	ES412798	Fumarylacetoacetase	0.76	0.015
contig_832	NP_495863*	Phenylalanine hydroxylase	1.47	0.005
contig_762	NP_499089*	Aspartyl- tRNA synthetase	1.18	0.031
contig_1024	NP_497078*	Glutathione peroxidase	1.25	0.007
contig_1164	NP_501914*	Valine-tRNA ligase	1.19	0.029
149396192	ES739601	Histidine-tRNA ligase	1.22	0.021
**Carbohydrate metabolism (17)**				
contig_2070	NP_500340*	Pyruvate dehydrogenase	1.24	0.024
149396498	ES739907	Methylmalonyl-CoA decarboxylase	0.71	0.008
156780356	EX008261	Methylmalonyl-CoA decarboxylase	0.72	0.015
156780356	EX008261	Propionyl-CoA carboxylase	0.72	0.015
158948398	EX910617	Phosphoglycolate phosphatase	1.18	0.009
145971363	ES411051	Citrate (Si)-synthase	0.67	0.010
145972833	ES412521	Citrate (Si)-synthase	0.70	0.045
156779893	EX007798	Acetoacetyl-CoA reductase	0.77	0.030
156786769	EX014674	Aconitate hydratase	0.64	0.038
149397746	ES741155	Aconitate hydratase	0.83	0.043
149397487	ES740896	Glucose-6-phosphate isomerase	0.75	0.044
115342257	EG025510	Fructose-bisphosphate aldolase	0.54	0.036
149400678	ES744087	Fructose-bisphosphate aldolase	0.60	0.049
145971975	ES411663	Protein-tyrosine-phosphatase	0.87	0.007
contig_2231	XP_790483*	Sorbitol dehydrogenase	1.30	0.046
contig_540	Q8HXX4*	Acetyl-CoA C-acetyltransferase	1.34	0.011
145970127	ES409815	Aconitate hydratase	0.78	0.019
**Nucleotide metabolism (9)**				

156779991	EX007896	DNA-directed RNA polymerase	0.78	0.011
149400704	ES744113	AICAR formyltransferase	0.58	0.042
145972757	ES412445	Ribose-phosphate diphosphokinase	0.76	0.004
156782981	EX010886	Ribose-phosphate diphosphokinase	0.81	0.003
145968881	ES408569	Pyruvate kinase	0.86	0.007
156783533	EX011438	Adenylosuccinate lyase	0.79	0.001
149398352	ES741761	Bis(5'-nucleosyl)-tetraphosphatase	1.33	0.035
156784077	EX011982	Nucleoside-triphosphatase	1.29	0.049
156780887	EX008792	Beta-ureidopropionase	0.89	0.029
**Metabolism of cofactors and vitamins (6)**				
149395588	ES739189	Formate-tetrahydrofolate ligase	0.88	0.003
149398099	ES741508	Methylenetetrahydrofolate reductase	1.23	0.026
115342189	EG025442	NADH dehydrogenase (ubiquinone)	1.83	0.007
145971869	ES411557	NADH dehydrogenase (ubiquinone)	1.52	0.007
156782379	EX010284	NADH dehydrogenase (ubiquinone)	0.78	0.023
156786466	EX014371	Acetolactate synthase	0.63	0.028
**Lipid metabolism (9)**				
145971440	ES411128	Aldehyde dehydrogenase (NAD+)	0.86	0.010
149396422	ES739831	Acyl-CoA dehydrogenase	0.82	0.005
183182916	FF679618	Trans-2-enoyl-CoA reductase (NADPH)	0.86	0.046
156785500	EX013405	Sphingomyelin phosphodiesterase	1.27	0.016
145974076	ES413764	Sphingomyelin phosphodiesterase	1.34	0.023
156785811	EX013716	Glycerol-3-phosphate dehydrogenase	0.85	0.038
contig_2386	NP_492417*	Fatty acid synthase	0.74	0.025
158950797	EX913393	Choline kinase	0.81	0.033
145971668	ES411356	NADPH-cytochrome P450	0.88	0.019
**Metabolism of other amino acids (5)**				
149401243	ES744652	Selenide, water dikinase	1.11	0.015
156783063	EX010968	Adenosylhomocysteinase	0.79	0.047
158952481	EX914791	Methionine adenosyltransferase	0.78	0.027
156781630	EX009535	Glutamate-cysteine ligase	1.25	0.012
156784452	EX012357	Ribonucleoside-diphosphate reductase	1.18	0.028
**Xenobiotic biodegradation and metabolism (3)**				
145971662	ES411350	Enoyl-CoA hydratase	0.85	0.030
145970639	ES410327	Acetyl-CoA C-acetyltransferase	1.17	0.003
156779520	EX007425	Glutaryl-CoA dehydrogenase	0.81	0.002
**Biosynthesis of secondary metabolites (3)**				
contig_2884	ABF95280*	dTDP-4-dehydro rhamnose reductase	1.12	0.006
145974196	ES413884	Glucose-1-phosphate thymidylyltransferase	0.73	0.005
156780031	EX007936	Hexokinase	1.23	0.046
**Glycan biosynthesis and metabolism (1)**				
Contig_864	XP_001676681*	Man(9)-alpha-mannosidase	0.81	0.014

[1]According to student t-test; P<0.05.

*Homolog accession number.
