# Supplementary material for: Transcriptional profiling of trait deterioration in the insect pathogenic nematode Heterorhabditis bacteriophora
Source: BMC Genomics. 2009 Dec 15;10:609. doi: 10.1186/1471-2164-10-609 (PMC2805696; doi:10.1186/1471-2164-10-609)
Supplement: Additional file 5 — Gene-specific primer sequences used for quantitative reverse transcription-PCR analysis. Primers were designed by aligning the EST sequences with their putative homologue from GenBank. [file 1471-2164-10-609-S5.PDF]

Additional file 5- List of gene-specific primer sequences used for quantitative real-time PCR analysis.

Target gene	Abbreviated name	Accession number	Forward sequence (5' to 3')	Reverse sequence (3' to 5')	Amplicon size
Yeast sec homolog	*Hb-sec-23*	EX012534	AAGTTGGTTTGAGCCACGAT	TTGCCTTACAACTGGCCTTT	318
Cytochrome c oxidase II	*Hb-co-II*	EX007863	GGTGAACCCCGTTTGTTAGA	AGCACCACAAATCTCAGAACA	221
Dauer overexpression	*Hb-dao-3*	ES739189	TGATCAAGTTGCCCGATACA	TCTCACGCGTTAAACCATCA	202
Uncoordinated (UNC-68)	*Hb-unc-68*	ES743277	AGTACGAGCTGGTGGAGGAA	AAGGCATCGATGATCAAACC	266
Aspartyl protease	*Hb-asp-3*	EX009021	TTTGGGTTCCATGCAAAAAT	CAGGCGAAACCTTGTGTTTT	214
High temperature-induced dauer formation	*Hb-hid-1*	EX010983	TCCGAATTCCTCAAAGAAGG	TGCATCCGTAATGTGGAAAA	270
Fatty acid desaturase	*Hb-fat-2*	ES744357	CAACTGTGGATGACGTGAGG	ACAATCATGTCCGACCACAA	224
Abnormal dauer formation	*Hb-daf-21*	EX007741	AGGAGCCTCAGTCACATGCT	ACGAGATGACGCAGAGATCA	186
RAB family- 33	*Hb-rab-33*	ES411895	ATGCTGGTGTAGGGAAAACG	GCTCCTGTCCTGCAGTATCC	147
Sphingosine-1-phosphate lyase	*Hb-spl-1*	FF679861	TGGAACCCCCTTTTACACTG	GAATGGCATTTGGGTTTTGT	311
Nitric oxide synthase interacting protein	*Hb-NOSIP*	ES412752	AAATTTGCTGCATTGGAAGG	TTTCCCTCATTACCGATTGC	176
Serine threonine kinase (UNC-51)	*Hb-unc-51*	FF681332	CTTGTCCTGGCAAACCAAGT	AGCAGAACCTTCGTCAGCAT	224
60S ribosomal protein	*Hb-60S-RP*	ES743844	AGGGAGCTTCGTGCTAAACA	GCTGAGGGAGAGCAACATTC	218
HRS-1 protein	*Hb-hrs-1*	ES739601	AACTCGGAGATGGAGAGCAA	CTCCACCCCGATCTTGAATA	244
Cyclophilin-1	*Hb-cyn-1*	EX007037	AAACCAGCAGGCAAAATCAC	CACCCCGCATCATAAATCG	172
Aldehyde dehydrogenase family member	Hb-alh-1	ES411128	CTTTAGGCGTTGTCGGTGTT	TGCTTCACCACAAACGAGAG	215
